# Advanced Platelet‐Rich Fibrin in Surgical Management of Medication‐Related Osteonecrosis of the Jaw: A Prospective Case Series Study

**DOI:** 10.1002/cre2.70346

**Published:** 2026-04-17

**Authors:** Besim Hajdari, Lavdie Leci Morina, Granit Gashi, Gloriosa Dobra, Besir Salihu, Venera Bimbashi

**Affiliations:** ^1^ Department of Maxillofacial Surgery, Faculty of Medicine, School of Dentistry University of Prishtina Prishtina Kosovo; ^2^ Department of Oral Surgery, Faculty of Medicine, School of Dentistry University of Prishtina Prishtina Kosovo; ^3^ Private Dental Clinic Ars – Medica Prishtina Kosovo; ^4^ Department of Pediatrics University Clinical Centre of Kosovo Prishtina Kosovo; ^5^ Faculty of Medicine University of Prishtina Prishtina Kosovo; ^6^ Department of Prosthodontics University Dentistry Clinical Centre of Kosovo Prishtina Kosovo

**Keywords:** medication‐related osteonecrosis of the jaw, oral surgery, platelet‐rich fibrin, regenerative medicine, wound healing

## Abstract

**Background:**

Medication‐related osteonecrosis of the jaw (MRONJ) is a severe complication associated with antiresorptive or antiangiogenic therapy. Surgical debridement is the mainstay of treatment for moderate to advanced disease, while adjunctive regenerative approaches such as advanced platelet‐rich fibrin (A‐PRF) may enhance soft tissue healing. This study aimed to evaluate the clinical outcomes of A‐PRF as an adjunct to surgical management of MRONJ.

**Methods:**

Seventeen patients diagnosed with MRONJ according to AAOMS criteria were prospectively enrolled. All patients underwent surgical removal of necrotic bone followed by placement of A‐PRF membranes and tension‐free mucosal closure. A‐PRF was prepared from autologous venous blood using a low‐speed centrifugation protocol. Mean follow‐up was 11.6 months. Treatment success was defined as complete mucosal healing without clinical signs of infection or bone exposure.

**Results:**

The mean age was 60.2 ± 7.8 years; 70.6% were female and 70.6% had cancer‐related disease. Stage 2 MRONJ was most prevalent (58.8%). Complete mucosal healing was achieved in 13 patients (76.5%). Four patients (23.5%) showed persistent bone exposure but reported symptomatic improvement.

**Conclusion:**

Adjunctive use of A‐PRF in MRONJ surgery was associated with favorable soft tissue healing and clinical improvement. Larger controlled studies are needed to confirm these findings.

**Abbreviations**
AAOMS: American Association of Oral and Maxillofacial Surgeons
APCs: autologous platelet concentrates
A‐PRF: advanced platelet‐rich fibrin
MRONJ: medication‐related osteonecrosis of the jaw
ORN: osteoradionecrosis
PRF: platelet‐rich fibrin

## 1 Introduction

Medication‐related osteonecrosis of the jaw (MRONJ) is a severe adverse effect associated with antiresorptive and antiangiogenic medications, characterized by persistent exposed bone, nonhealing intraoral or extraoral fistulae, or pathological fractures lasting more than 8 weeks in patients without a history of radiation therapy to the jaws or metastatic disease (Ruggiero et al. 2014). Although osteonecrosis of the jaws may arise from different etiologies, including radiation‐induced injury (osteoradionecrosis, ORN), trauma, or idiopathic causes, MRONJ represents a distinct clinical entity with unique pharmacological, biological, and pathological mechanisms (Zadik et al. 2021; Marx 2003; Miyoshi et al. 2024; Lončar Brzak et al. 2023). Unlike ORN, which primarily results from radiation‐induced vascular damage, MRONJ is driven by medication‐mediated suppression of bone remodeling and impairment of tissue healing (Lončar Brzak et al. 2023; Akashi et al. 2018; Henien et al. 2016; Lončar Brzak et al. 2019).

According to the American Association of Oral and Maxillofacial Surgeons (AAOMS), MRONJ is staged from “at risk” to stage 3 based on clinical and radiographic features (Ruggiero et al. 2022). Despite extensive research, the pathophysiology of MRONJ remains incompletely understood and is considered multifactorial. Reduced mandibular vascularity, local trauma, dental infection, bacterial colonization, and impaired immune response have all been implicated as contributing factors (van Gemert et al. 2018; Wimalawansa 2008). The risk of MRONJ increases with cumulative drug dose, duration of therapy, and potency of antiresorptive agents, particularly intravenous bisphosphonates and denosumab, compared with oral formulations (Miyoshi et al. 2024; Wimalawansa 2008; Marx et al. 2005; Lasseter et al. 2005; Valente et al. 2019; Kün‐Darbois and Fauvel 2021). These drugs induce osteoclast apoptosis, suppress bone turnover, and interfere with osteoblast, fibroblast, and keratinocyte function, thereby compromising both hard‐ and soft‐tissue healing (Valente et al. 2019; Coffman et al. 2021). Furthermore, nitrogen‐containing bisphosphonates exert direct cytotoxic effects on oral mucosal cells and exhibit long‐term skeletal retention due to their high affinity for hydroxyapatite (Wimalawansa 2008; Marx et al. 2005; Lasseter et al. 2005; Valente et al. 2019; Coffman et al. 2021; Omi and Mishina 2022; Byrne et al. 2024).

Currently, there is no universally accepted gold‐standard treatment protocol for MRONJ, and management is guided primarily by disease stage and symptom severity (Lončar Brzak et al. 2019; AlDhalaan et al. 2020; Sarkarat et al. 2022). Conservative approaches, including systemic antibiotics, antiseptic mouth rinses, analgesics, and optimization of oral hygiene, are generally recommended in early stages and aim to control infection and pain rather than achieve complete resolution (Sarkarat et al. 2022; Bacci et al. 2022). However, conservative therapy alone rarely results in predictable mucosal healing. Surgical intervention, consisting of sequestrectomy, debridement, or resection of necrotic bone, has demonstrated higher success rates, particularly for stage 2 and stage 3 MRONJ, and is currently endorsed by AAOMS as the preferred treatment approach for advanced disease (Lončar Brzak et al. 2019; Pereira‐Silva et al. 2024; Zheng et al. 2023). Nevertheless, even surgical management may be associated with delayed healing or recurrence (Sánchez‐Gallego Albertos et al. 2021a), highlighting the need for adjunctive regenerative strategies. Among biological adjuvants, autologous platelet concentrates (APCs)—including platelet‐rich plasma (PRP), platelet‐rich fibrin (PRF), and plasma rich in growth factors (PRGF)—have gained increasing attention due to their capacity to deliver growth factors that promote angiogenesis, inflammation modulation, soft‐tissue repair, and bone regeneration (Maracineanu et al. 2025; Inchingolo et al. 2023; Del Fabbro et al. 2015; Choukroun et al. 2006; Bennardo et al. 2023). Several clinical studies have reported enhanced mucosal closure and symptomatic improvement when PRF membranes are applied following surgical debridement in MRONJ lesions (Wimalawansa 2008; Bennardo et al. 2023). However, reported outcomes remain heterogeneous, largely because of differences in preparation protocols, centrifugation parameters, and study design.

Advanced platelet‐rich fibrin (A‐PRF) represents a modified PRF formulation produced using low‐speed centrifugation, resulting in increased leukocyte content and a more sustained release of growth factors such as VEGF, PDGF, and TGF‐β (Del Fabbro et al. 2015). These biological properties suggest that A‐PRF may offer advantages over conventional PRF in supporting wound healing and tissue regeneration. Despite its growing application in oral and maxillofacial surgery, clinical evidence specifically evaluating A‐PRF as an adjunct in MRONJ management remains limited.

Therefore, the objective of this prospective clinical study was to evaluate the clinical efficacy of advanced platelet‐rich fibrin (A‐PRF) as an adjunct to surgical debridement in patients with MRONJ, with particular emphasis on mucosal healing, symptom resolution, and overall treatment success across different disease stages.

## 2 Materials and Methods

### 2.1 Study Design and Setting

This study was designed as a prospective descriptive clinical case series to evaluate the clinical outcomes and feasibility of advanced platelet‐rich fibrin (A‐PRF) as an adjunct to surgical management of medication‐related osteonecrosis of the jaw (MRONJ). No control or comparator group was included. Patients were consecutively enrolled between March 2024 and March 2025 at the Department of Oral Surgery, University Dentistry Clinical Center of Kosovo (UDCCK). The study was conducted in accordance with the Declaration of Helsinki (1975, revised 2013). Ethical approval was obtained from the Institutional Ethics Committee of the University Dentistry Clinical Center of Kosovo (Approval No: 648‐2/2021). Written informed consent was obtained from all participants prior to enrollment and surgical intervention.

### 2.2 Patient Selection and Clinical Evaluation

Only patients diagnosed with MRONJ were included in this study. Osteoradionecrosis (ORN) and other causes of jaw osteonecrosis were not enrolled. MRONJ diagnosis was established according to the American Association of Oral and Maxillofacial Surgeons (AAOMS) criteria:

I. current or previous treatment with antiresorptive and/or antiangiogenic agents,
II. exposed bone or bone that can be probed through an intraoral or extraoral fistula persisting for more than 8 weeks, and
III. no history of radiation therapy to the jaws or metastatic disease to the jaws.

Inclusion criteria:

- Diagnosis of MRONJ (AAOMS Stage 1–3)
- History of antiresorptive and/or antiangiogenic therapy
- Patients with osteoporosis or malignant disease
- Ability and willingness to undergo surgical treatment with A‐PRF and attend follow‐up visits

Exclusion criteria:

- Uncontrolled systemic disease (e.g., uncontrolled diabetes mellitus, severe immunodeficiency)
- Pregnancy or lactation
- Active malignant tumor involving the jaws or jaw metastases
- Contraindications to surgery

All patients underwent comprehensive clinical and radiographic evaluation, including panoramic radiographs and/or cone beam computed tomography (CBCT), to determine the extent and stage of jaw osteonecrosis.

### 2.2 A‐PRF Preparation Protocol and Surgical Procedure

**Figure 1:**
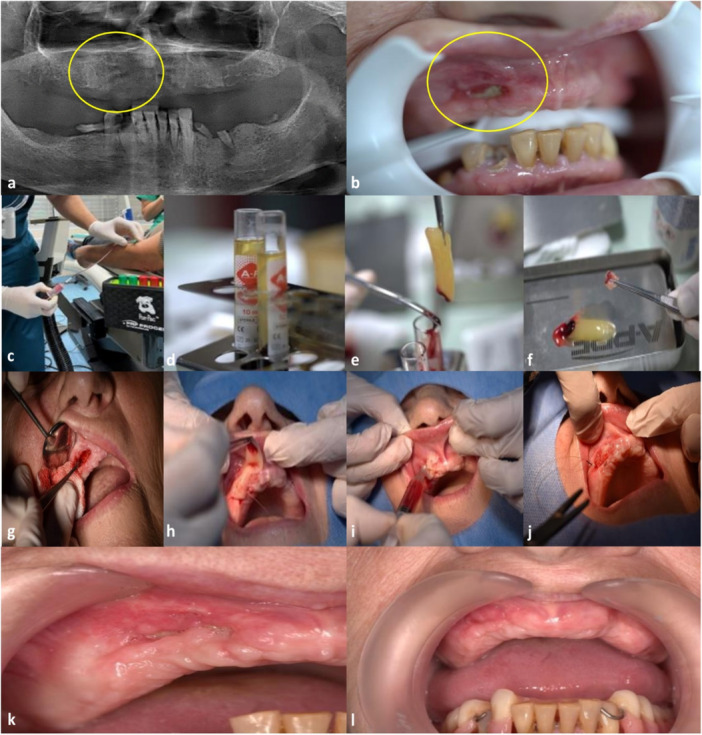
Case 4. A 69‐year‐old woman. (a) Orthopantomogram (OPG) obtained prior to A‐PRF application. (b) Nonhealing osteonecrotic lesion located in the right anterior maxillary region. (c–f) Preparation of A‐PRF: venous blood collection, A‐PRF tube after centrifugation, fibrin clot formation, and shaping of the A‐PRF clot. (g) Surgical resection of infected and necrotic tissues. (h) Placement of the shaped A‐PRF clot and coverage with a PRF membrane. (i) Initial application of liquid PRF. (j) Wound closure with sutures. (k) Healed surgical site 1 month after the first A‐PRF application. (l) Stable healing observed 1 year after the first A‐PRF application.

Immediately prior to surgery, 10 mL of peripheral venous blood was collected from each patient into sterile, dry glass tubes without anticoagulant. Samples were centrifuged at 1300 rpm for 14 min using a dedicated A‐PRF centrifuge (PROCESS for PRF, Nice, France), following the protocol described by Choukroun et al. (2014). The time from venipuncture to start of centrifugation did not exceed 60 s. After centrifugation, the fibrin clot was separated from the red blood cell fraction and gently compressed in a sterile PRF box to obtain A‐PRF membranes and plugs. Only A‐PRF was used throughout the study. (Figure 1C–F.

All procedures were performed under local anesthesia with 2% lidocaine containing 1:100,000 epinephrine, without sedation.

*Step 1–Flap design and access*

A full‐thickness mucoperiosteal flap was elevated using a crestal incision with one or two vertical releasing incisions as needed to obtain adequate access and flap mobility.

*Step 2––Debridement and sequestrectomy*

Necrotic bone was removed using rotary instruments and surgical curettes until viable bone was reached. Viable bone was defined intraoperatively as bone exhibiting punctate bleeding upon gentle drilling or curettage and showing a firm, nonfriable texture. Sharp bony edges were rounded, and the surgical field was thoroughly irrigated with sterile saline.

*Step 3–A‐PRF application*

One to two layers of A‐PRF membrane were placed directly over the exposed viable bone, completely covering the defect. Additional A‐PRF plugs were used to fill deeper defects when present.

*Step 4–Flap advancement and wound closure*

The flap was repositioned to achieve tension‐free primary closure, confirmed by passive flap adaptation without resistance. When required, periosteal releasing incisions were performed to facilitate advancement. Closure was achieved using 4‐0 resorbable sutures (Vicryl®) in interrupted or mattress technique, depending on defect size and flap tension. No additional grafting materials or barrier membranes were used.

*Step 5–Postoperative adjunctive therapy*

Patients received amoxicillin/clavulanic acid 875/125 mg twice daily for 7 days (or clindamycin in allergic patients), 0.12% chlorhexidine mouth rinse twice daily for 14 days, and analgesics as needed.

### 2.3 Follow‐Up and Outcome Evaluation

Patients were strictly followed clinically at 1 week, 1 month, 3 months, and 6 months postoperatively. A 12‐month and 24‐month follow‐up was optional for patients who were followed up for an extended period.

The primary outcome was complete clinical resolution of MRONJ following surgical treatment with A‐PRF membranes. Complete resolution (“cured”) was operationally defined as:

- Complete mucosal coverage of the surgical site
- Absence of exposed bone
- Absence of pain, swelling, suppuration, or fistula
- No clinical signs of infection

The secondary outcome was persistent disease, defined as:

- Ongoing bone exposure and/or
- Persistence of symptoms (pain, swelling, suppuration, or fistula) at the last follow‐up visit.

Clinical outcomes were assessed through standardized intraoral examination performed at follow‐up visits.

Additional recorded outcome‐related variables included:

- Time to last follow‐up (months)
- Changes in presenting clinical signs (e.g., exposed bone, pain, purulence)
- Relationship between outcome and MRONJ stage

Outcome categories were dichotomized as:

- Cured (complete resolution)
- Persistent exposure (non‐healed)

### 2.4 Statistical Analysis

Statistical analysis was performed using descriptive and inferential methods. Continuous variables (age, duration of antiresorptive therapy, follow‐up time) were summarized using mean ± standard deviation and ranges. Categorical variables (sex, diagnosis, antiresorptive drug, MRONJ stage, first clinical sign, outcome) were summarized as frequencies and percentages.

Associations between clinical outcome (cured vs persistent exposure) and categorical variables such as MRONJ stage, antiresorptive medication type, and primary diagnosis were explored using the chi‐square test or Fisher′s exact test when appropriate.

Comparisons of continuous variables between outcome groups were performed using the independent samples t‐test. The level of statistical significance was set at *p* < 0.05.

## 3 Results

Seventeen patients diagnosed with medication‐related osteonecrosis of the jaw (MRONJ) were included and treated surgically with adjunctive application of advanced platelet‐rich fibrin (A‐PRF). The mean age was 60.2 ± 7.8 years (range: 49–74), and most patients were female (*n* = 12, 70.6%). Twelve patients (70.6%) had an underlying oncological diagnosis, most commonly breast cancer (*n* = 5, 29.4%), followed by prostate cancer (*n* = 2, 11.8%), lung cancer (*n* = 2, 11.8%), multiple myeloma (*n* = 2, 11.8%), and other malignancies (renal cell carcinoma, colon carcinoma, and maxillary squamous cell carcinoma; *n* = 3, 17.6%). Five patients (29.4%) were receiving antiresorptive therapy for osteoporosis (Table 2). Zoledronate was the most frequently administered antiresorptive agent (*n* = 11, 64.7%), followed by pamidronate (n = 3, 17.6%), ibandronate (*n* = 2, 11.8%), and alendronate (*n* = 1, 5.9%). The mean duration of antiresorptive therapy was 51.6 months (range: 23–95 months). MRONJ lesions were predominantly located in the posterior mandible, often involving molar regions and, in several cases, multiple adjacent sites (Tables 1 and 2).

**Table 1:** Clinical characteristics, treatment details, and outcomes of patients with medication‐related osteonecrosis of the jaw (MRONJ) treated surgically with advanced platelet‐rich fibrin (A‐PRF) membranes.

Pt.	Age	Sex	Diagnosis	Antiresorptive	Duration (mo)	ONJ location	Stage	First clinical sign	Outcome	Follow up (mo)
1	62	M	Prostate Ca	Zoledronate H	25	11,12	2	Painful exposed bone	Cured	12
2	56	F	Breast & Lung Ca	Zoledronate H	23	22,23	2	Purulent discharge	Cured	9
3	49	F	Multiple Myeloma	Pamidronate IV	27	25	2	Delayed socket healing	Cured	16
4	69	F	Colon Ca	Zoledronate H	72	13,12	3	Bone exposure, sequestrum + halitosis	Cured	12
5	63	F	Osteoporosis + HTA	Zoledronate H	28	45,34	2	Swelling and pain	Cured	11
6	63	M	Renal Cell Ca	Zoledronate H	33	27	2	Exposed necrotic bone	Cured	7
7	48	F	Breast Ca	Zoledronate H	39	35,36,37	2	Fistula/sinus tract	Cured	9
8	58	F	Breast Ca	Zoledronate H	53	48	2	Pain on mastication	Cured	13
9	57	F	Osteoporosis	Ibandronate H	71	33	1	Asymptomatic exposure	Cured	12
10	74	F	Osteoporosis	Alendronate L	95	45	1	Rough bone sensation	Cured	14
11	67	F	Breast Ca + Liver Mets	Zoledronate H	60	26,27,28	3	Sequestrum with exposed necrotic Bone	Cured	6
12	52	F	Breast Ca	Zoledronate H	56	47	1	Nonhealing extraction	Exposed	15
13	55	F	Osteoporosis	Ibandronate PO + IV	63	38	2	Inflammation, pain, Nonhealing extraction	Cured	24
14	52	M	Lung Ca	Pamidronate IV	31	23,24	2	Bone exposure	Cured	17
15	66	M	Prostate Ca, Diabetes	Zoledronate H	66	45,46,47	3	Extraoral fistula with purulence	Exposed	6
16	62	F	SCC Maxilla	Zoledronate H	48	33	2	Exposed bone + swelling	Cured	12
17	70	M	Multiple Myeloma	Pamidronate H (IV)	84	46,47,48	3	Exposed bone, purulence and swelling	Exposed	5

**Table 2:** Patient characteristics summary.

Characteristic	Value
Total patients (*n*)	17
Mean age ± SD (years)	60.2 ± 7.8
Sex distribution	Female: 12 (70.6%) Male: 5 (29.4%)
Primary diagnosis	Cancer: 12 (70.6%)
	Osteoporosis: 5 (29.4%)
* Breast cancer*	5 (29.4%)
* Prostate cancer*	2 (11.8%)
* Lung cancer*	2 (11.8%)
* Multiple myeloma*	2 (11.8%)
* Other (renal, colon, SCC)*	3 (17.6%)
Antiresorptive drug used	Zoledronate H: 11 (64.7%)
	Pamidronate IV: 3 (17.6%)
	Ibandronate: 2 (11.8%)
	Alendronate: 1 (5.9%)
Mean duration of antiresorptive therapy	51.6 months (range: 23–95 months)
Most common ONJ locations	Posterior mandible (e.g., 33–48)
ONJ Stage distribution (AAOMS)	Stage 1: 3 (17.6%)
	Stage 2: 10 (58.8%)
	Stage 3: 4 (23.5%)
Most common first clinical signs	Exposed bone: 6 (35.3%)
	Pain/swelling: 5 (29.4%)
	Suppuration/fistula: 4 (23.5%)
	Asymptomatic: 2 (11.8%)
Outcome	Cured: 13 (76.5%)
	Persistent exposure: 4 (23.5%)
Mean follow‐up duration	11.6 months (range: 5–24 months)

According to the AAOMS staging system, 10 patients (58.8%) were classified as Stage 2, four (23.5%) as Stage 3, and three (17.6%) as Stage 1 (Tables 1 and 2). The most frequent initial clinical manifestation was exposed bone (*n* = 6, 35.3%), followed by pain or swelling (*n* = 5, 29.4%), suppuration or fistula formation (*n* = 4, 23.5%), and asymptomatic exposure (*n* = 2, 11.8%). Patients with Stage 3 disease typically exhibited more advanced clinical features, including sequestrum formation, extensive necrotic bone exposure, and, in some cases, extraoral fistulas or oroantral communication.

All patients underwent surgical debridement of necrotic bone followed by placement of A‐PRF membranes and tension‐free mucosal closure. After a mean follow‐up of 11.6 months (range: 5–24 months), complete mucosal healing was achieved in 13 patients (76.5%), who were classified as cured, while four patients (23.5%) exhibited persistent bone exposure at the last follow‐up (Tables 1 and 2).

When outcomes were analyzed according to MRONJ stage, all Stage 2 lesions demonstrated complete healing (10/10, 100%), compared with 2/3 (66.7%) Stage 1 lesions and 2/4 (50%) Stage 3 lesions (Table 3). Although a trend toward lower healing rates with increasing disease stage was observed, the association between MRONJ stage and treatment outcome did not reach statistical significance (χ² test, *p* = 0.063).

**Table 3:** Association between MRONJ stage and treatment outcome.

MRONJ stage	Cured *n* (%)	Persistent exposure *n* (%)	Total
Stage 1	2 (66.7%)	1 (33.3%)	3
Stage 2	10 (100%)	0 (0%)	10
Stage 3	2 (50%)	2 (50%)	4
Total	14	3	17

*Note:* Chi‐square test: *p* = 0.063.

Analysis according to primary diagnosis revealed cure rates of 76.9% (10/13) among oncological patients and 100% (4/4) among patients treated for osteoporosis (Table 4). This difference was not statistically significant (χ² test, *p* = 0.757).

**Table 4:** Association between primary diagnosis and treatment outcome.

Diagnosis	Cured *n* (%)	Persistent exposure *n* (%)	Total
Cancer	10 (76.9%)	3 (23.1%)	13
Osteoporosis	4 (100%)	0 (0%)	4
Total	14	3	17

*Note:* Chi‐square test: *p* = 0.757.

Mean age did not differ significantly between patients who achieved complete healing and those with persistent exposure (59.6 vs. 62.7 years, respectively; independent samples t‐test, *p* = 0.551) (Table 5).

**Table 5:** Comparison of mean age between outcome groups.

Outcome group	Mean age (years)
Cured	59.6
Persistent exposure	62.7

*Note:* Independent samples *t*‐test: *p* = 0.551.

Detailed evaluation of the four patients with persistent bone exposure showed that two cases were classified as Stage 3 MRONJ and presented with extensive mandibular involvement accompanied by sequestrum formation and/or extraoral fistula. Both had received long‐term intravenous bisphosphonate therapy and had advanced malignant disease, with additional systemic comorbidities such as diabetes mellitus or metastatic disease. The remaining two patients had Stage 1 and Stage 3 lesions, respectively, and had a history of prolonged antiresorptive exposure. In all four cases, partial mucosal coverage and symptom reduction were observed postoperatively; however, small, stable areas of exposed bone persisted beyond 8 weeks. None of these patients exhibited clinical progression or required reoperation during the observation period.

**Figure 2:**
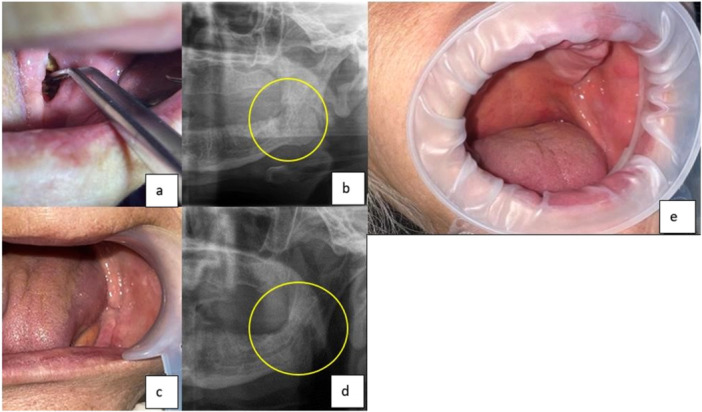
Follow‐up of a 55‐year‐old female (Case 13) with Stage 2 MRONJ. (a) Nonhealing socket 4 months postextraction. (b) Preoperative OPG. (c) Healed site 2 weeks after A‐PRF application. (d) OPG at 1‐year follow‐up. (e) Fully healed mucosa after 1 year.

**Figure 3:**
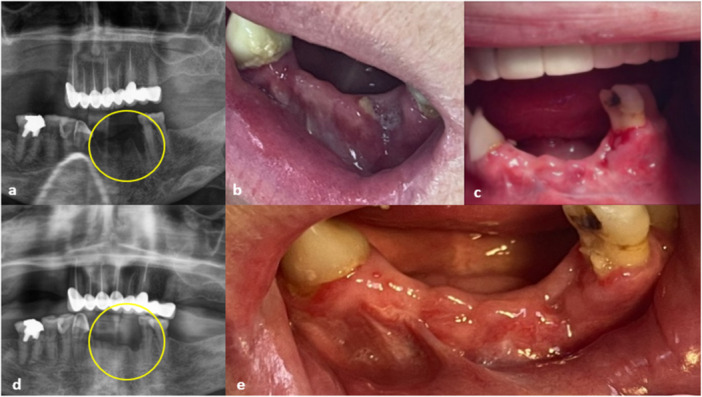
Case 16, 62‐year‐old woman, (a) OPG before application of A‐PRF, (b) the wound after 5 months after the extraction of her lower left canine, (c) the healed wound after 2 weeks of application of A‐PRF, (d) the OPG after 1 year of A‐PRF application, and (e) the healed wound after 1 year of application of A‐PRF.

Overall, surgical debridement combined with A‐PRF membrane application resulted in high rates of mucosal healing across MRONJ stages. While advanced stage and greater systemic burden appeared to be associated with less favorable outcomes, these associations did not reach statistical significance in this cohort. Figures 2 and 3 illustrate the clinical course of a representative Stage 2 MRONJ patient demonstrating complete mucosal healing following surgical debridement and A‐PRF application.

## 4 Discussion

This prospective clinical case series study investigated the adjunctive use of advanced platelet‐rich fibrin (A‐PRF) in the surgical treatment of MRONJ and achieved complete mucosal closure in 76.5% of the cases. This result aligns with previously published outcomes for other autologous platelet concentrates (APCs) (Inchingolo et al. 2023; Del Fabbro et al. 2015; Choukroun et al. 2006; Bennardo et al. 2023). Recent systematic reviews further support these findings, reporting wide but generally favorable healing ranges for both PRP and PRF in MRONJ management (Ferreira et al. 2025; Fortunato et al. 2020; Nowak et al. 2024). For instance, Bennardo et al (Bennardo et al. 2023). documented success rates of 87.3% with PRGF and 86.9% with l‐PRF. Although our healing percentage was slightly lower, it remains within a comparable range, particularly given the prospective design and standardized surgical approach employed in the present study. Ferreira et al. reported complete mucosal healing rates ranging from 36% to 100% for PRF‐based protocols, concluding that platelet concentrates may enhance healing, bone regeneration, and symptom relief when used adjunctively (Ferreira et al. 2025). In contrast to the variability commonly reported in PRGF and l‐PRF investigations (Nowak et al. 2024; Roman et al. 2024; Adamska et al. 2025; Cano‐Durán et al. 2017; Alrmali et al. 2023) regarding patient staging, surgical protocols, and outcome definitions, our research applied consistent criteria and included only individuals diagnosed according to the AAOMS classification (Lončar Brzak et al. 2019). Studies such as those by (Sánchez‐Gallego Albertos et al. 2021b) and (Mozzati et al. 2012) reported even higher recovery rates with PRGF (up to 87%); however, their heterogeneous interventions and inconsistently defined recurrence or partial‐healing parameters limit the generalizability of those findings. Similarly, l‐PRF studies by Giudice et al (Giudice et al. 2020a). and Yuce et al (Yüce et al. 2021). demonstrated no major long‐term differences compared to conventional surgery, though they indicated potential short‐term benefits. This observation is consistent with the conclusions of (Fortunato et al. 2020), who found no statistically significant differences between APC‐assisted surgery and surgery alone for MRONJ treatment, despite an overall trend toward clinical improvement. Our results also revealed symptomatic improvement, even in four patients who retained areas of bone exposure, implying that A‐PRF may provide palliative advantages beyond complete mucosal closure.

Importantly, our clinical series encompassed a wide range of MRONJ severities, including advanced Stage 3 lesions, whereas many comparative studies primarily evaluated Stages 1–2 (Nørholt and Hartlev 2016; Szentpeteri et al. 2020). The favorable healing trends observed in this study could be associated with the biological properties of A‐PRF, including its high content of platelets, leukocytes, and growth factors. However, given the absence of a matched control group treated with surgical debridement alone, the precise contribution of A‐PRF to these outcomes cannot be determined. Therefore, the results should be interpreted as indicative rather than confirmatory evidence of A‐PRF efficacy.

A recent meta‐analysis focusing on l‐PRF in ONJ management demonstrated a pooled complete resolution rate of 94.3%, with low heterogeneity, suggesting that PRF‐based approaches, alone or combined with other therapies, are associated with high rates of lesion resolution (Muñoz‐Salgado et al. 2023) A‐PRF represents a refinement of the original PRF protocol, designed to enhance the regenerative capabilities of the fibrin matrix (Chmielewski et al. 2025). (Ghanaati et al. 2014) showed that lower centrifugation speeds and shorter spin durations yield prolonged release of growth factors and improved cellular content, characterized by elevated leukocyte and platelet concentrations that promote both soft‐ and hard‐tissue repair. Comparable findings were reported by (Kobayashi et al. 2016) and (Masuki et al. 2016), who observed that A‐PRF exhibits higher total protein and growth‐factor release over time than conventional PRF. Additionally, A‐PRF membranes display more uniform cellular distribution and an increased proportion of neutrophilic granulocytes, potentially enhancing antimicrobial and wound‐healing responses (Masuki et al. 2016; Giudice et al. 2020b). These biological advantages are consistent with the mechanistic explanations proposed in recent systematic reviews, which highlight the anti‐inflammatory, angiogenic, and immunomodulatory roles of platelet concentrates in MRONJ wound environments (Ferreira et al. 2025; Nowak et al. 2024). Collectively, these results provide biological justification for integrating A‐PRF into MRONJ surgical protocols and may underlie the positive outcomes documented in our cohort.

While our focus was primarily on mucosal and clinical recovery, the influence of A‐PRF on bone remodeling also merits attention. Prior randomized controlled trials have explored the modified formulation A‐PRF + , which has been linked to improvements in bone density, particularly when combined with graft materials (Giudice et al. 2020b; Kalash et al. 2017). Studies such as those by (Clark et al. 2018) and (Alhaj et al. 2018). indicated trends toward better bone quality following A‐PRF+ use, although most differences were not statistically significant. Similarly, Nowak et al. reported that APCs did not achieve statistically significant superiority in treating established MRONJ, despite an overall treatment success estimate of approximately 83%, underscoring the ongoing uncertainty regarding their isolated therapeutic effect (Nowak et al. 2024). In MRONJ management, where infection control and mucosal closure are key objectives, the anti‐inflammatory and regenerative attributes of A‐PRF may contribute more prominently to soft‐tissue healing than to quantifiable gains in mineral density.

### 4.1 Limitations and Future Directions

Despite the encouraging outcomes observed in this prospective study, several limitations should be acknowledged. First, the sample size was relatively small (*n* = 17), which may limit the statistical power and generalizability of the findings. Although our study included a diverse patient cohort with varying disease stages and systemic conditions, larger multicenter trials are needed to validate the reproducibility of these results across broader populations. Second, the absence of a control group limits our ability to isolate the specific effect of A‐PRF. Without direct comparison to surgical debridement alone, we cannot determine whether the improved outcomes were solely attributable to A‐PRF or to the standardized surgical management itself.

4.1.1

Additionally, variations in patients′ systemic conditions, concurrent medications, and antiresorptive drug holidays were not uniformly controlled, which may have influenced healing trajectories. Standardizing these variables in future investigations could help clarify their role in treatment success. Future research should explore the molecular mechanisms underlying the regenerative effects of A‐PRF, particularly its influence on angiogenesis, immune modulation, and soft tissue remodeling in MRONJ lesions. Long‐term follow‐up beyond 24 months would also be beneficial for assessing the stability of mucosal healing and potential recurrence rates. Therefore, the findings should be interpreted as exploratory and hypothesis‐generating, supporting the need for well‐designed randomized controlled trials to clarify the role of A‐PRF in MRONJ management.

## 5 Conclusions

This prospective clinical case series suggests that the adjunctive use of advanced platelet‐rich fibrin (A‐PRF) in the surgical management of MRONJ is associated with a favorable trend toward mucosal healing; however, in the absence of a control group, definitive conclusions regarding its efficacy cannot be drawn. A‐PRF may serve as a supportive adjunct to surgical debridement by promoting soft‐tissue healing through its biological properties. Larger, well‐designed randomized controlled trials with standardized protocols are required to clarify the specific contribution of A‐PRF in MRONJ treatment.

## Author Contributions

Besim Hajdari, Lavdie Leci Morina, Venera Bimbashi, and Granit Gashi: conceptualization. Besir Salihu, Lavdie Leci Morin, and Granit Gashi: methodology and formal analysis. Besim Hajdari, Lavdie Leci Morina, and Besir Salihu: writing—original draft preparation. Besim Hajdari, Lavdie Leci Morina, Granit Gashi, Venera Bimbashi, and Gloriosa Dobra: writing—review and editing. All authors have read and approved the final version of the manuscript.

## Funding

The authors have nothing to report.

## Ethics Statement

Ethical approval was obtained from the Ethics Committee, University Dentistry Clinical Centre of Kosova, registration number: 648/2‐2021. All procedures conducted in studies involving human accomplices were in accordance with the ethical standards of the institutional and/or national research committee, as well as the 1975 Helsinki Declaration, revised in 2013.

## Consent

The patients obtained written informed consent for publishing the information and photographs in the journal.

## Conflicts of Interest

The authors declare no conflicts of interest.

## Data Availability

All original contributions presented in this study are included in the article. For any further inquiries, please contact the corresponding author.

## References

[cre270346-bib-0001] Adamska, P. , M. Stasiak , N. Kobusińska , M. Bartmański , A. Zedler , and M. Studniarek . 2025. “Treatment of Medication‐Related Osteonecrosis of the Jaw Without and With the Use of Advanced Platelet‐Rich Fibrin: A Retrospective Clinical Study.” Journal of Functional Biomaterials 16, no. 5: 180. 10.3390/jfb16050180. 40422844 PMC12112225

[cre270346-bib-0002] Akashi, M. , S. Wanifuchi , E. Iwata , et al. 2018. “Differences Between Osteoradionecrosis and Medication‐related Osteonecrosis of the Jaw.” Oral and Maxillofacial Surgery 22, no. 1: 59–63. 10.1007/s10006-017-0667-5. 29224060

[cre270346-bib-0003] AlDhalaan, N. A. , A. BaQais , and A. Al‐Omar . 2020. “Medication‐Related Osteonecrosis of the Jaw: A Review.” Cureus 12, no. 2: e6944. 10.7759/cureus.6944. 32190495 PMC7067354

[cre270346-bib-0004] Alhaj, F. , M. Shokry , and N. Attia . 2018. “The Efficiency of Using Advanced Platelet Rich Fibrin–autogenous Bone Graft Mixture Around Immediately Placed Dental Implants in Mandibular Molar Region: (randomized Controlled Clinical Trial).” Egyptian Dental Journal 64: 2023–2035. 10.21608/edj.2018.76743.

[cre270346-bib-0005] Alrmali, A. , M. H. A. Saleh , S. M. S. Kurdi , H. Sabri , M. M. Meghil , and H. L. Wang . 2023. “Prevention and Management of Drug‐induced Osteonecrosis of the Jaws Using Platelet‐rich Fibrin: A Clinical Feasibility Study.” Clinical and Experimental Dental Research 9, no. 5: 791–798. 10.1002/cre2.775. 37605488 PMC10582237

[cre270346-bib-0007] Bacci, C. , A. Cerrato , E. Bardhi , A. C. Frigo , S. A. Djaballah , and S. Sivolella . 2022. “A Retrospective Study on the Incidence of Medication‐related Osteonecrosis of the Jaws (mronj) Associated With Different Preventive Dental Care Modalities.” Supportive care in cancer: official journal of the Multinational Association of Supportive Care in Cancer 30, no. 2: 17231729. 10.1007/s00520-021-06587-x. PMC8727393 34580783

[cre270346-bib-0008] Bennardo, F. , L. Gallelli , C. Palleria , et al. 2023. “Can Platelet‐rich Fibrin Act As A Natural Carrier For Antibiotics Delivery? A Proof‐of‐concept Study For Oral Surgical Procedures.” BMC Oral Health 23, no. 1: 134. 36894902 10.1186/s12903-023-02814-5 PMC9996939

[cre270346-bib-0009] Byrne, H. , S. O'Reilly , C. S. Weadick , P. Brady , and R. N. Ríordáin . 2024. “How We Manage Medication‐related Osteonecrosis of the Jaw.” European Journal of Medical Research 29: 402. 10.1186/s40001-024-01912-6. 39095845 PMC11297747

[cre270346-bib-0010] Cano‐Durán, J. A. , J. F. Peña‐Cardelles , D. Ortega‐Concepción , V. M. Paredes‐Rodríguez , M. García‐Riart , and J. López‐Quiles . 2017. “The Role of Leucocyte‐rich and Platelet‐rich Fibrin (lprf) in the Treatment of the Medication‐related Osteonecrosis of the Jaws (MRONJ).” Journal of Clinical and Experimental Dentistry 9, no. 8: 1051. 10.4317/jced.54154. PMC5601107 28936298

[cre270346-bib-0011] Chmielewski, M. , A. Pilloni , and P. Adamska . 2025. “Advanced Platelet‐rich Fibrin Plus (a‐prf+) As An Additive To Hard Tissue Managing Protocols in Oral Surgery: A Systematic Review.” Journal of Functional Biomaterials 16, no. 4: 145. 10.3390/jfb16040145. 40278253 PMC12028204

[cre270346-bib-0012] Choukroun, J. , A. Diss , A. Simonpieri , et al. 2006. “Platelet‐Rich Fibrin (PRF): A Second‐generation Platelet Concentrate. Part Iv: Clinical Effects on Tissue Healing.” Oral Surgery, Oral Medicine, Oral Pathology, Oral Radiology, and Endodontology 101, no. 3: e56–e60. 10.1016/j.tripleo.2005.07.011. 16504852

[cre270346-bib-0013] Clark, D. , Y. Rajendran , S. Paydar , et al. 2018. “Advanced Platelet‐rich Fibrin and Freeze‐dried Bone Allograft For Ridge Preservation: A Randomized Controlled Clinical Trial.” Journal of Periodontology 89: 379–387. 10.1002/JPER.17-0466. 29683498 PMC6483085

[cre270346-bib-0014] Coffman, A. A. , J. Basta‐Pljakic , R. M. Guerra , et al. 2021. “A Bisphosphonate With A Low Hydroxyapatite Binding Affinity Prevents Bone Loss in Mice After Ovariectomy and Reverses Rapidly With Treatment Cessation.” JBMR Plus 5, no. 4: e10476. 10.1002/jbm4.10476. 33869992 PMC8046044

[cre270346-bib-0015] Del Fabbro, M. , G. Gallesio , and M. Mozzati . 2015. “Autologous Platelet Concentrates For Bisphosphonate‐related Osteonecrosis of the Jaw Treatment and Prevention. A Systematic Review of the Literature.” European Journal of Cancer 51, no. 1: 62–74. 10.1016/j.ejca.2014.10.015. 25466505

[cre270346-bib-0016] Ferreira, F. , C. Faria , and D. H. Pozza . 2025. “Autologous Platelet Concentrates in the Management of Medication‐Related Osteonecrosis of the Jaw: A Systematic Review.” Medicina 61, no. 8: 1496. 10.3390/medicina61081496. 40870542 PMC12388798

[cre270346-bib-0017] Fortunato, L. , F. Bennardo , C. Buffone , and A. Giudice . 2020. “Is the Application of Platelet Concentrates Effective in the Prevention and Treatment of Medication‐related Osteonecrosis of the Jaw? A Systematic Review.” Journal of Cranio‐Maxillofacial Surgery 48, no. 3: 268–285. 10.1016/j.jcms.2020.01.014. 32063481

[cre270346-bib-0018] van Gemert, J. T. M. , J. H. Abbink , R. J. J. van Es , A. J. W. P. Rosenberg , R. Koole , and E. M. Van Cann . 2018. “Early and Late Complications in the Reconstructed Mandible With Free Fibula Flaps.” Journal of Surgical Oncology 117, no. 4: 773–780. 10.1002/jso.24976. 29448299 PMC5901040

[cre270346-bib-0019] Ghanaati, S. , P. Booms , A. Orlowska , et al. 2014. “Advanced Platelet‐rich Fibrin: A New Concept For Cell‐based Tissue Engineering By Means of Inflammatory Cells.” Journal of Oral Implantology 40, no. 6: 679–689. 24945603 10.1563/aaid-joi-D-14-00138

[cre270346-bib-0020] Giudice, A. , A. Antonelli , and M. D. Fortunato . 2020b. “L. Usefulness of Advanced‐platelet Rich Fibrin (A‐PRF) and Injectable‐platelet Rich Fibrin (i‐PRF) in the mAnagement of A Massive Medication‐related Osteonecrosis of the Jaw (MRONJ): a 5‐Years Follow‐up Case Report.” Indian Journal of Dental Research 31, no. 5: 813–818. 33433526 10.4103/ijdr.IJDR_689_19

[cre270346-bib-0021] Giudice, A. , A. Antonelli , D. Muraca , and L. Fortunato . 2020a. “Usefulness of Advanced‐platelet Rich Fibrin (A‐PRF) and Injectable‐platelet Rich Fibrin (i‐PRF) in the Management of A Massive Medication‐related Osteonecrosis of the Jaw (MRONJ): a 5‐year follow‐up case report.” Indian Journal of Dental Research 31, no. 5: 813. 33433526 10.4103/ijdr.IJDR_689_19

[cre270346-bib-0022] Henien, M. , V. Patel , C. Sproat , and M. McGurk . 2016. “Spontaneous Osteonecrosis of the Maxilla.” Dental Update 43, no. 6: 563–566. 10.12968/denu.2016.43.6.563. 29148651

[cre270346-bib-0023] Inchingolo, A. M. , G. Malcangi , I. Ferrara , et al. 2023. “MRONJ Treatment Strategies: A Systematic Review and Two Case Reports.” Applied Sciences 13, no. 7: 4370. 10.3390/app13074370.

[cre270346-bib-0024] Kalash, S. , N. Aboelsaad , M. Shokry , and J. Choukroun . 2017. “The Efficiency of Using Advanced Prfxenograft Mixture Around Immediate Implants in the Esthetic Zone: A Randomized Controlled Clinical Trial.” Journal of Osseointegration 9: 317–322. 10.23805/jo.2017.09.04.02.

[cre270346-bib-0025] Kobayashi, E. , L. Flückiger , M. Fujioka‐Kobayashi , et al. 2016. “Comparative Release of Growth Factors From PRP, PRF, and Advanced‐PRF.” Clinical Oral Investigations 20, no. 9: 2353–2360. 26809431 10.1007/s00784-016-1719-1

[cre270346-bib-0026] Kün‐Darbois, J. D. , and F. Fauvel . 2021. “Medication‐Related Osteonecrosis and Osteoradionecrosis of the Jaws: Update and Current Management.” Morphologie 105, no. 349: 170–187. 10.1016/j.morpho.2020.11.008. 33281055

[cre270346-bib-0027] Lasseter, K. C. , A. G. Porras , A. Denker , A. Santhanagopal , and A. Daifotis . 2005. “Pharmacokinetic Considerations in Determining the Terminal Elimination Half‐lives of Bisphosphonates.” Clinical Drug Investigation 25, no. 2: 107–114. 10.2165/00044011-200525020-00003. 17523760

[cre270346-bib-0028] Lončar Brzak, B. , L. Horvat Aleksijević , E. Vindiš , et al. 2023. “Osteonecrosis of the Jaw.” Dentistry Journal 11, no. 1: 23. 10.3390/dj11010023. 36661560 PMC9858620

[cre270346-bib-0029] Lončar Brzak, B. , V. Vučičević Boras , A. Kotarac Knežević , M. Sušić , S. Seiwerth , and D. Gabrić . 2019. “Idiopathic Exposed Bone Lesions of the Jaw.” Dentistry Journal 7, no. 2: 55. 10.3390/dj7020055. 31159353 PMC6630877

[cre270346-bib-0030] Maracineanu, R. , A. Tudor , I. Hum , et al. 2025. “Platelet‐Rich Fibrin in MRONJ Management: A Prospective Comparative Study on Its Effectiveness in Prevention and Treatment.” Medicina 61, no. 4: 625. 10.3390/medicina61040625. 40282916 PMC12028908

[cre270346-bib-0031] Marx, R. E. 2003. “Pamidronate (Aredia) and zoledronate (Zometa) induced avascular necrosis of the jaws: A growing epidemic.” Journal of Oral and Maxillofacial Surgery 61, no. 9: 1115–1117. 10.1016/s0278-2391(03)00720-1. 12966493

[cre270346-bib-0032] Marx, R. E. , Y. Sawatari , M. Fortin , and V. Broumand . 2005. “Bisphosphonate‐iNduced Exposed Bone (osteonecrosis/osteopetrosis) of the Jaws: Risk Factors, Recognition, Prevention, and Treatment.” Journal of Oral and Maxillofacial Surgery 63, no. 11: 1567–1575. 10.1016/j.joms.2005.07.010. 16243172

[cre270346-bib-0033] Masuki, H. , T. Okudera , T. Watanebe , et al. 2016. “Growth Factor and Pro‐inflammatory Cytokine Contents in Platelet‐rich Plasma (PRP), Plasma Rich in Growth Factors (PRGF), Advanced Platelet‐rich Fibrin (A‐PRF), and Concentrated Growth Factors (CGF).” International Journal of Implant Dentistry 2, no. 1: 19. 27747711 10.1186/s40729-016-0052-4 PMC5005757

[cre270346-bib-0034] Miyoshi, T. , M. Otsuru , K. Morishita , et al. 2024. “Differences Between Medication‐related Osteonecrosis of the Jaw Caused By Bisphosphonates and Denosumab: Histological, Molecular Biological, and Clinical Studies.” Cureus 16, no. 6: e62855. 10.7759/cureus.62855. 39036251 PMC11260442

[cre270346-bib-0035] Mozzati, M. , G. Gallesio , V. Arata , R. Pol , and M. Scoletta . 2012. “Platelet‐Richtherapies in the Treatment of Intravenous Bisphosphonate‐related Osteonecrosis of the Jaw: A Report of 32 Cases.” Oral Oncology 48, no. 5: 469–474. 22265335 10.1016/j.oraloncology.2011.12.004

[cre270346-bib-0036] Muñoz‐Salgado, A. , F. Silva , M. Padín‐Iruegas , et al. 2023. “Leukocyte and Platelet Rich Fibrin in the Management of Medication‐related Osteonecrosis of the Jaw: A Systematic Review and Meta‐analysis.” Medicina Oral Patología Oral y Cirugia Bucal 28, no. 4: e317–e329. 10.4317/medoral.25733. 36641740 PMC10314351

[cre270346-bib-0037] Nowak, S. M. , R. Sacco , F. L. Mitchell , V. Patel , and K. Gurzawska‐Comis . 2024. “The Effectiveness of Autologous Platelet Concentrates in Prevention and Treatment of Medication‐related Osteonecrosis of the Jaws: A Systematic Review.” Journal of Cranio‐Maxillofacial Surgery 52, no. 6: 671–691. 10.1016/j.jcms.2024.01.007. 38644092

[cre270346-bib-0038] Nørholt, S. E. , and J. Hartlev . 2016. “Surgical Treatment of Osteonecrosis of the Jaw With the Use of Platelet‐rich Fibrin: A Prospective Study of 15 Patients.” International Journal of Oral and Maxillofacial Surgery 45, no. 10: 1256–1260. 10.1016/j.ijom.2016.04.010. 27179556

[cre270346-bib-0039] Omi, M. , and Y. Mishina . 2022. “Roles of Osteoclasts in Alveolar Bone Remodeling.” Genesis 60, no. 8–9: e23490. 10.1002/dvg.23490. 35757898 PMC9786271

[cre270346-bib-0041] Pereira‐Silva, M. , H. Hadad , L. K. de Jesus , et al. 2024. “Ozone Therapy Effect in Medication‐related Osteonecrosis of the Jaw As Prevention Or Treatment: Microtomographic, Confocal Laser Microscopy and Histomorphometric Analysis.” Clinical Oral Investigations 28, no. 2: 151. 10.1007/s00784024-05547-z. 38360985

[cre270346-bib-0042] Roman, C. , M. A. Moldovan , L. S. Pop , S. Megieșan , and C. I. Faur . 2024. “Platelet‐Rich Fibrin Treatment Evaluation in Patients With Medication‐related Osteonecrosis of the Jaw and Osteoradionecrosis.” Journal of Clinical Medicine 13, no. 12: 3473. 10.3390/jcm13123473. 38930013 PMC11204677

[cre270346-bib-0043] Ruggiero, S. L. , T. B. Dodson , T. Aghaloo , E. R. Carlson , B. B. Ward , and D. Kademani . 2022. “American Association of Oral and Maxillofacial Surgeons′ Position Paper on Medication‐related Osteonecrosis of the Jaws—2022 Update.” Journal of Oral and Maxillofacial Surgery 80, no. 5: 920–943. 10.1016/j.joms.2022.02.008. 35300956

[cre270346-bib-0044] Ruggiero, S. L. , T. B. Dodson , J. Fantasia , et al. 2014. “American Association of Oral and Maxillofacial Surgeons Position Paper on Medication‐related Osteonecrosis of the Jaw—2014 Update.” Journal of Oral and Maxillofacial Surgery: Official Journal of the American Association of Oral and Maxillofacial Surgeons 72, no. 10: 1938–1956. 10.1016/j.joms.2014.04.031. 25234529

[cre270346-bib-0045] Sánchez‐Gallego Albertos, C. , J. L. Del Castillo Pardo de Vera , A. Viejo Llorente , and J. L. Cebrián Carretero . 2021a. “Medication Related Osteonecrosis of the Jaws (MRONJ): Factors Related To Recurrence After Treatment With Surgery and Platelet Rich Plasma (PRP) Placement.” Medicina Oral, Patologia Oral Y Cirugia Bucal 26, no. 6: e684–e690. 10.4317/medoral.24007. 34704981 PMC8601641

[cre270346-bib-0046] Sánchez‐Gallego Albertos, C. , D. C. Pardo , J. L. de Vera , L. A. Viejo , and J. L. Cebrián Carretero . 2021b. “Medication Related Osteonecrosis of thejaws (MRONJ): Factors Related To Recurrence After Treatment Withsurgery and Platelet Rich Plasma (PRP) Placement.” Med Oral PatolOral Cir Bucal 26, no. 6: e684–e690. 10.4317/medoral.24007 PMC8601641 34704981

[cre270346-bib-0047] Sarkarat, F. , A. Modarresi , A. Riyahi , P. Mortazavi , F. Tabandeh , and V. Rakhshan . 2022. “Efficacy of Hyaluronic Acid, Absorbable Collagen Sponge, and Their Combination in Minimizing Bisphosphonate‐related Osteonecrosis of the Jaws (bronj) After Dental Extraction: A Preliminary Animal Histomorphometric Study.” Maxillofacial Plastic and Reconstructive Surgery 44, no. 1: 8. 10.1186/s40902-022-00337-7. 35230522 PMC8888787

[cre270346-bib-0048] Szentpeteri, S. , L. Schmidt , L. Restar , G. Csaki , G. Szabo , and M. Vaszilko . 2020. “The Effect of Plateletrich Fibrin Membrane in Surgical Therapy of Medication‐related Osteonecrosis of the Jaw.” Journal of Oral and Maxillofacial Surgery 78, no. 5: 738–748. 10.1016/j.joms.2019.12.008. 31945309

[cre270346-bib-0049] Valente, N. A. , S. Chatelain , F. Alfonsi , C. Mortellaro , and A. Barone . 2019. “Medication‐Related Osteonecrosis of the Jaw: The Use of Leukocyte‐platelet‐rich Fibrin As An Adjunct in the Treatment.” Journal of Craniofacial Surgery 30, no. 4: 1095–1101. 10.1097/SCS.0000000000005475. 30908443

[cre270346-bib-0050] Wimalawansa, S. J. 2008. “Insight Into Bisphosphonate‐associated Osteomyelitis of the Jaw: Pathophysiology, Mechanisms and Clinical Management.” Expert Opinion on Drug Safety 7, no. 4: 491–512. 10.1517/14740338.7.4.491. 18613812

[cre270346-bib-0051] Yüce, M. O. , E. Adalı , and G. Işık . 2021. “The effect of Concentrated Growth Factor (CGF) in the Surgical Treatment of Medication‐related Osteonecrosis of the Jaw (MRONJ) in Osteoporosis Patients: A Randomized Controlled Study.” Clinical Oral Investigations 25, no. 7: 4529–4541. 33392802 10.1007/s00784-020-03766-8

[cre270346-bib-0052] Zadik, Y. , Y. Ganor , O. Rimon , E. Bersudski , and A. Meirovitz . 2021. “Assessment of Jaw Osteonecrosis Diagnostic Criteria in Cancer Patients With A History of Radiation Therapy and Exposure To Bone‐modifying Agents.” Radiotherapy and Oncology 156: 275–280. 10.1016/j.radonc.2020.12.026. 33373641

[cre270346-bib-0053] Zheng, Y. , X. Dong , S. Chen , et al. 2023. “Low‐Level Laser Therapy Prevents Medication‐related Osteonecrosis of the Jaw‐like Lesions via IL‐1RA‐Mediated Primary Gingival Wound Healing.” BMC Oral Health 23, no. 1: 14. 10.1186/s12903-02202678-1. 36627695 PMC9832759

